# Supplementary material for: Chlorobaculum tepidum outer membrane vesicles may transport biogenic elemental sulfur
Source: Appl Environ Microbiol. 2025 Jun 18;91(7):e01019-25. doi: 10.1128/aem.01019-25 (PMC12285230; doi:10.1128/aem.01019-25)
Supplement: Supplemental material — Supplemental methods, Figures S1 to S4, and Tables S1 and S2. [file aem.01019-25-s0001.pdf]

SUPPLEMENTAL MATERIAL

METHODS AND MATERIALS

Protein Extraction and Shotgun Proteomic Analysis of *Cba. tepidum* S(0) Globules

A comprehensive analysis of extraction conditions (1) developed the protein extraction method used here. S(0) globules were suspended in 100 mM triethylammonium bicarbonate (TEAB) with 2% w/v CHAPS detergent followed by sonication on ice (2 min increments, 10 mins total) and centrifugation (16,000 × g, 30 mins) to pellet S(0). The supernatant was removed and treated with DetergentOUT™ spin columns (G-Biosciences) followed by concentration in a SpeedVac™ at 37 °C (Thermo Fisher Scientific Inc.). The dried pellet was resuspended in 0.5 M TEAB with 0.1% w/v SDS and reduced by treatment with tris-(2-carboxyethyl)phosphine (TCEP, 4.3 mM, 60 °C, 1 hr). Free cysteines were blocked with methyl methane-thiosulfonate (8.3 mM, 10 mins, room temperature). Proteins were digested overnight (37 °C) with 1 µg trypsin (Promega) per 10 µg protein. Digestion was stopped by adding formic acid to 1.5% v/v. Peptides were cleaned up prior to LC-MS/MS using Bond Elut OMIX C18 tips (Agilent) according to manufacturer instructions, and concentrated to dryness using a SpeedVac™ vacuum concentrator at 37 °C. Dried peptides were stored at -80 °C.

Peptides were separated by low pH reverse phase high performance liquid chromatography on a Tempo LC-MALDI spotter (Eksigent). Peptides were resolubilized in Mobile Phase A (2% acetonitrile, 0.1% trifluoroacetic acid (TFA), loaded onto a 1.2 µl CapRod 18E capillary column (Merck KGaA), and washed with 10 column volumes of 98% Mobile Phase A/2% Mobile Phase B (98% acetonitrile, 0.1% trifluoroacetic acid). Peptides were eluted by an 80 min gradient to 55% acetonitrile and 0.1% TFA. Eluate was spotted in 10 second increments onto stainless steel target plates with α-cyano-4-hydroxycinnamic acid (Sigma-Aldrich) as matrix.

Peptides were identified by MALDI-TOF/TOF mass spectrometry in positive ion reflector mode on an AB Sciex 5800 MALDI-TOF/TOF Analyzer. MS data were collected over a mass range of 800-4000 m/z and were processed with internal calibration. A maximum of 15 precursors, with signal-to-noise ratios of at least 20, were selected per spot for MS/MS. Fragmentation was induced with 1 kV collision energy. MS/MS spectra were processed with default calibration and submitted for Mascot v2.4 database searchers through Protein Pilot software v4.5 (ABSciex). Spectra were searched against an NCBI database of the *C. tepidum* proteome (downloaded October 29, 2014). Peptide identifications with 95% confidence or greater and protein identifications containing at least one significant unique peptide were accepted.

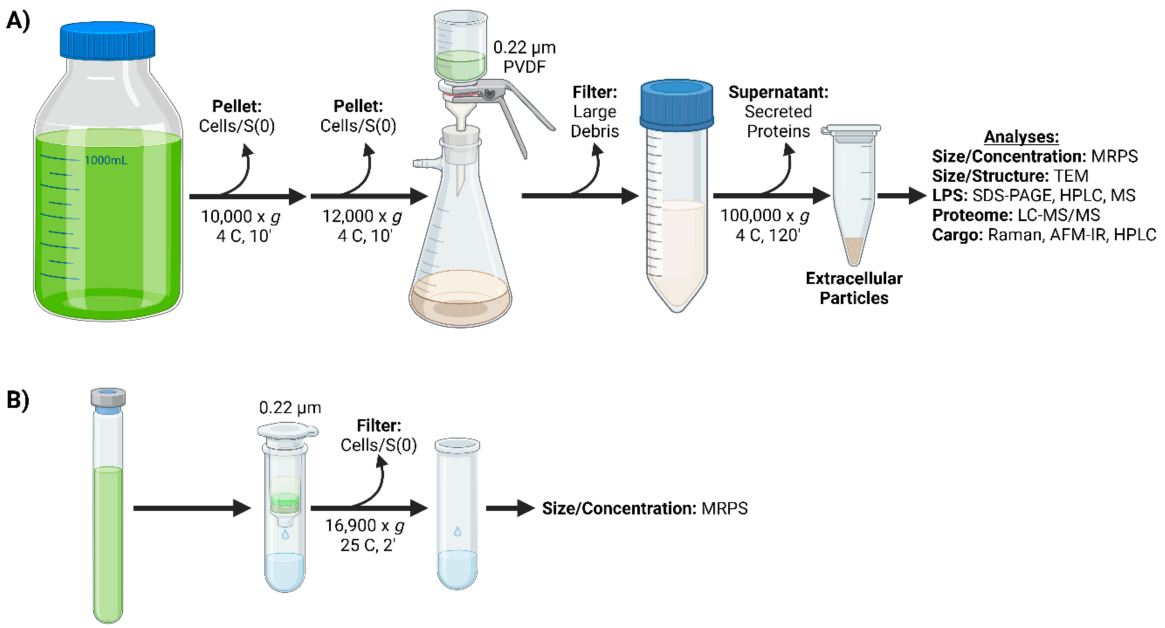

Figure S1. Workflow for particle purification methods from *Cba. tepidum* cultures. A) Extracellular particles for analyses requiring large amounts of input material were prepared by differential centrifugation. B) Extracellular particle samples for routine counting and sizing were prepared by centrifugation through a 0.22 µm filter. Non-standard abbreviations: MRPS-microfluidic resistive pulse sensing; AFM-IR-atomic force microscopy with Infrared spectroscopy. Created in BioRender https://BioRender.com/q133wb4

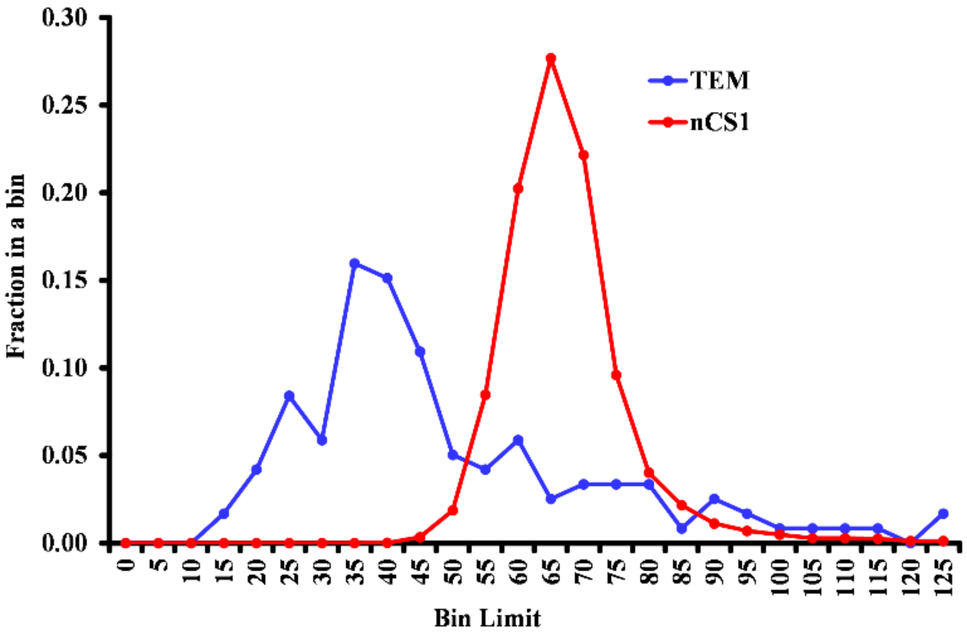

Figure S2. Particle size distributions by TEM and MRPS

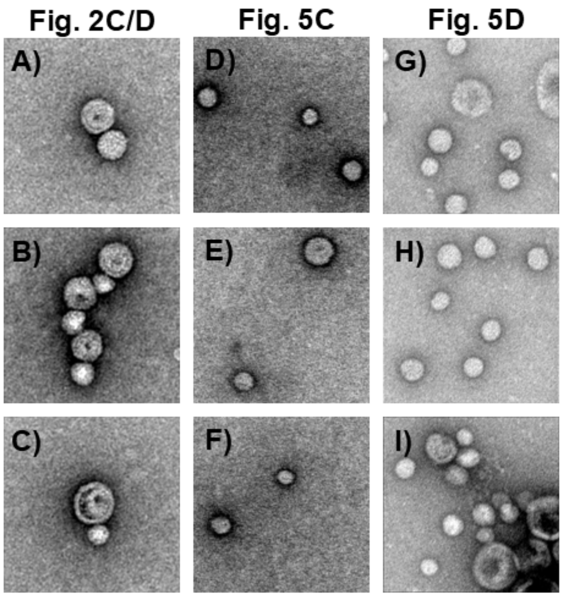

Figure S3. Close up images of OMVs from TEM images used in Figures 2 (A-C) and 5 (D-I). Each image in this collection is 200 nm on each side and were produced by selecting, copying and scaling regions of interest from the initial images in ImageJ. No changes were made to any other image parameters.

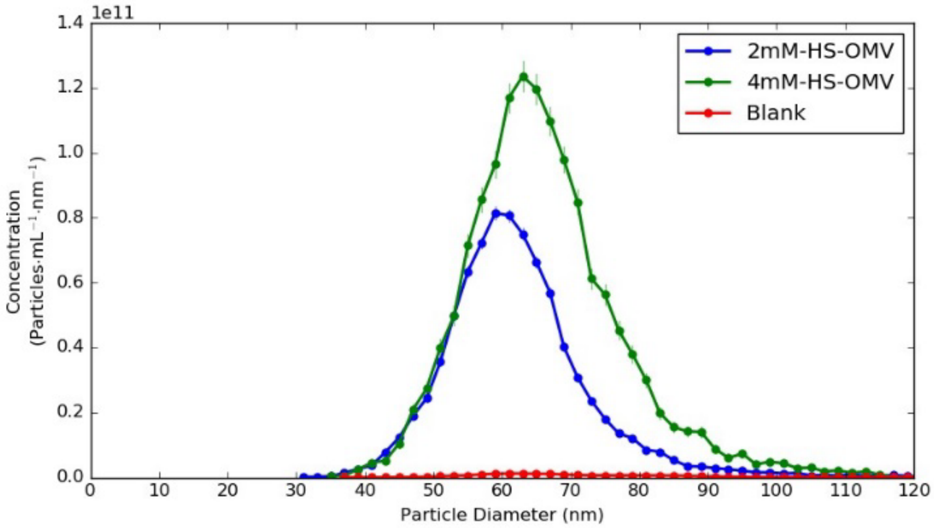

Figure S4. Example of raw nCS1 particle count data

Table S1. Proteins identified by shotgun proteomic analysis of S(0) globules produced by wild type *Cba. tepidum*. Proteins also identified in our gel-based studies (1, 2) or in OMV proteomic studies from the literature are indicated.

Protein Information						Detected in:			
Locus Tag	Accession[1]	Gene Name	Description	Predicted Location [2]	COG Cat.[3]	S(0) shotgun	S(0) gel	Cells gel	OMVs Reference
CT0018	NP_660924	*atpH*	ATP synthase F0F1 subunit delta	C	C	X			10
CT0020	NP_660926	*atpE*	ATP synthase F0 subunit C	CM#	C	X			9
CT0027	NP_660933		hypothetical protein CT0027	U	--	X			
CT0031	NP_660937	*ftsA*	cell division protein FtsA	C	D	X			9,10
CT0068	NP_660974		hemagglutinin-related protein	OM#	M		X		5,7
CT0089	NP_660995	*clpB-2*	ATP-dependent Clp protease, ATP-binding subunit ClpB	C	O	X		X	3,9,10
CT0131	NP_661037		hypothetical protein CT0131	C	R	X			
CT0150	NP_661056	*nusG*	transcription antitermination protein NusG	C	K	X			3,9
CT0153	NP_661059	*rplJ*	50S ribosomal protein L10	C#	J	X			4,9,10,12
CT0154	NP_661060	*rplL*	50S ribosomal protein L7/L12	U	J	X			4
CT0155	NP_661061	*rpoB*	DNA-directed RNA polymerase subunit beta	C	K	X		X	3,4,9-11
CT0156	NP_661062	*rpoC*	DNA-directed RNA polymerase subunit beta'	C	K	X			3,4,9-11
CT0159	NP_661065	*efp*	elongation factor P	C	J	X			9
CT0160	NP_661066	*hupB*	DNA-binding protein HU-beta	C	L	X	X	X	9-12
CT0173	NP_661079	*serB*	phosphoserine phosphatase	C	E	X			11
CT0249	NP_661153		glutathione S-transferase	C	E	X			
CT0254	NP_661158	*ompH*	outer membrane protein OmpH	U	M	X			4,5,13
CT0264	NP_661168	*rho*	transcription termination factor Rho	C	K	X			10
CT0288	NP_661192	*rpsA*	30S ribosomal protein S1	C	J	X			3,9,10
CT0302	NP_661206	*petC5*	cytochrome b6-f complex, iron-sulfur subunit	CM	C	X	X	X	3
CT0303	NP_661207	*petB*	cytochrome b-c complex, cytochrome b subunit	CM	C	X			3,9,10
CT0312	NP_661216		DnaK suppressor protein	C	T	X			
CT0350	NP_661254	*fabI*	enoyl-(acyl-carrier-protein) reductase	CM	I	X			3,12
CT0529	NP_661429	*groS* *groES*	co-chaperonin GroES	C	O	X		X	9
CT0530	NP_661430	*groL* *groEL*	chaperonin GroEL	C	O	X	X	X	3-5,8-13
CT0531	NP_661431		sensor histidine kinase/response regulator	CM	T	X			10,11
CT0547	NP_661447	*mreB-1*	rod shape-determining protein MreB	C	D	X			3,5,10

CT0563	NP_661463	*tyrS*	tyrosyl-tRNA synthetase	C	J	X			
CT0607	NP_661507		hypothetical protein CT0607	C	S	X			
CT0638	NP_661535		peptidoglycan-associated lipoprotein	OM	M	X			3,4,9-11,13
CT0642	NP_661539		hypothetical protein CT0642	C	--	X		X	
CT0643	NP_661540	*dnaK*	molecular chaperone DnaK	C	O	X	X	X	5,8-10
CT0644	NP_661541		HSP20 family protein	C	O	X	X	X	
CT0829	NP_661723	*htpG*	heat shock protein 90	C	O	X		X	9,10
CT0841	NP_661735	*trx-2*	thioredoxin	C	CO	X		X	
CT0893	NP_661786		hypothetical protein (Porin_5)	OM	--	X	X		11
CT0903	NP_661796		transcriptional regulator	C	P	X			
CT0941	NP_661834	*btuR, CobO, CobP*	cob(I)alamin adenosyltransferase	C	H	X			
CT0960	NP_661853	*purC*	phosphoribosylaminoimidazole-succinocarboxamide synthase	C	F	X			9,10
CT0980	NP_661873		ArsA ATPase	C	P	X			
CT1007	NP_661900		hypothetical protein CT1007 (DsrE/DsrF - like family)	CM	--	X			
CT1054	NP_661945	*prc*	carboxyl-terminal protease	CM	M	X			4,7-11
CT1133	NP_662024		hypothetical protein (CRISPR-associated protein)	U	--		X		
CT1170	NP_662061		hypothetical protein CT1170	C	I	X			9
CT1225	NP_662115		N-acetylmuramoyl-L-alanine amidase	U	V	X			9-11
CT1239	NP_662127	*secA*	preprotein translocase subunit SecA	C	U	X		X	3,9-11
CT1297	NP_662185	*bchI chlI*	magnesium-chelatase subunit I	C	R	X			
CT1309	NP_662197		hypothetical protein CT1309	U	--	X			
CT1353	NP_662240		OmpA family protein	OM	M	X		X	4,9-13
CT1361	NP_662248	*prs prsA*	ribose-phosphate pyrophosphokinase	C	EF	X			3,4,9,10
CT1362	NP_662249	*rplY ctc*	50S ribosomal protein L25 general stress protein	C	J	X			9,10
CT1447	NP_662333		serine protease	P	O	X	X	X	3,4,10,11,13
CT1485	NP_662370	*grpE*	heat shock protein GrpE	C	O	X			9
CT1499	NP_662384	*fmoA*	bacteriochlorophyll A protein	Csm	--	X		X	
CT1577	NP_662460	*frr*	ribosome recycling factor	C	J	X		X	9
CT1591	NP_662474	*ribBA*	3,4-dihydroxy-2-butanone 4-phosphate synthase	C	H	X			3
CT1649	NP_662532	*pnp*	polynucleotide phosphorylase/polyadenylase	C	J	X			3,5,9,10,12
CT1742	NP_662622	*feoB-1*	ferrous iron transport protein B	CM	P	X			

CT1743	NP_662623	*feoA-1*	ferrous iron transport protein A	U	P	X			
CT1744	NP_662624		hypothetical protein CT1744	U	P	X			
CT1745	NP_662625		hypothetical protein CT1745	U	--	X		X	
CT1780	NP_662659	*tsf*	elongation factor Ts	C	J	X			3,9,12
CT1781	NP_662660	*rpsB*	30S ribosomal protein S2	C	J	X			3,4,10,11,13
CT1782	NP_662661	*rpsI*	30S ribosomal protein S9	C	J	X			3,4,10,11,13
CT1785	NP_662664		ATP-binding Mrp/Nbp35 family protein	CM	D	X			3,4,10
CT1804	NP_662683		hypothetical protein	OM	--	X	X	X	
CT1833	NP_662712	*gatC*	aspartyl/glutamyl-tRNA amidotransferase subunit C	C	J	X			3
CT1867	NP_662744		hypothetical protein CT1867	P	S	X			
CT1921	NP_662798		cysteine synthase/cystathionine beta-synthase	C	E	X			8,9
CT1939	NP_662816		ArsA ATPase	C	P	X			
CT1942	NP_662819	*csmA*	chlorosome envelope protein A	Csm	--	X		X	
CT1943	NP_662820	*csmC*	chlorosome envelope protein C	Csm	--	X	X	X	
CT1947	NP_662824	*ssb-1*	single-strand binding protein	C	L	X			6,9
CT1955	NP_662832		magnesium-chelatase, bacteriochlorophyll c-specific subunit	C	H	X		X	
CT1970	NP_662846		HSP20 family protein	U	O	X	X	X	10
CT1986	NP_662862		hypothetical protein CT1986	U	R	X			
CT2001	NP_662877	*bcp-2, ahpC, tsa*	bacterioferritin comigratory protein, thiol peroxidase	C	O	X			8,9
CT2026	NP_662901		c-type cytochrome	U	--	X			
CT2033	NP_662908	*atpA*	ATP synthase F0F1 subunit alpha	C	C	X		X	3,5,8-10
CT2047	NP_662922		AcrB/AcrD/AcrF family protein	CM	V	X			10
CT2049	NP_662924		LipD protein, putative	OM	MU	X	X		4,11,13
CT2054	NP_662929	*csmB*	chlorosome envelope protein B	Csm	--	X		X	
CT2067	NP_662942		pentapeptide repeat-containing protein	E	S	X			
CT2097	NP_662971		hypothetical protein CT2097	U	S	X			
CT2101	NP_662975		hypothetical protein CT2101	C	S	X		X	
CT2129	NP_663003	*rplT*	50S ribosomal protein L20	C	J	X			4,9,10,12
CT2144	NP_663018		outer surface protein, putative	OM	M	X		X	5,12
CT2147	NP_663021		hypothetical protein CT2147	C	--	X			
CT2151	NP_663025	*bchB*	light-independent protochlorophyllide reductase subunit B	C	C	X			
CT2160	NP_663034	*gidB rsmG*	16S rRNA methyltransferase GidB	C	J	X			

CT2161	NP_663035	*rplQ*	50S ribosomal protein L17	C	J	X	X	4,9,10,12
CT2162	NP_663036	*rpoA*	DNA-directed RNA polymerase subunit alpha	C	K	X		3,9,10,12
CT2177	NP_663051	*rplE*	50S ribosomal protein L5	C	J	X		3,4,9,10,12,13
CT2182	NP_663056	*rplP*	50S ribosomal protein L16	C	J	X		4,9,12
CT2186	NP_663060	*rplB*	50S ribosomal protein L2	C	J	X		3,4,9,10,12,13
CT2191	NP_663065	*tuf*	elongation factor Tu	C	J	X		3,4,7-11,13
CT2215	NP_663089	*gatB*	aspartyl/glutamyl-tRNA amidotransferase subunit B	C	J	X		3,8
CT2216	NP_663090		hypothetical protein	CM	S	X		
CT2234	NP_663108	*atpD-2*	ATP synthase F0F1 subunit beta	C	C	X	X	3,5,8-10
CT2264	NP_663137	*surA, prsA*	peptidyl-prolyl cis-trans isomerase SurA	P	O	X		4,9,10,13
CT2281	NP_663152	*clpB-1*	ATP-dependent Clp protease, ATP-binding subunit ClpB	C	O	X		3,9,10

[1] RefSeq (NP_xxxxxx) or GenBank (AAYxxxxx) Accession

[2] Location prediction by PSORTb, except for except for manual annotations noted with #. Csm: chlorosome; C: cytoplasmic; CM: cytoplasmic membrane; OM: outer membrane; P: periplasmic; U: unknown.

[3] Abbreviations for COG functional categories are provided in Table S2.

Table S2. Functional classification of proteins associated with S0 based on COG categories, and portion of proteins within COG categories with homologs previously identified in proteomic studies of outer membrane vesicles.

COG Category		S^0 proteins		S^0 proteins with OMV protein homologs	
		count[1]	% of total[1]	count	% in COG category
C	Energy production and conversion	8	7.9%	6	75%
D	Cell cycle control, cell division, chromosome partitioning	3	3.0%	3	100%
E	Amino acid metabolism and transport	4	4.0%	3	75%
F	Nucleotide metabolism and transport	2	2.0%	2	100%
H	Coenzyme metabolism and transport	3	3.0%	1	33%
I	Lipid metabolism and transport	2	2.0%	2	100%
J	Translation, ribosomal structure and biogenesis	20	19.8%	18	90%
K	Transcription	5	5.0%	5	100%
L	Replication, recombination and repair	2	2.0%	2	100%
M	Cell wall/membrane/envelope biogenesis	7	6.9%	7	100%
O	Post-translational modification, protein turnover, chaperones	13	12.9%	11	85%
P	Inorganic ion transport and metabolism	6	5.9%	0	0%
R	General Functional Prediction only	3	3.0%	0	0%
S	Function Unknown	6	5.9%	0	0%
T	Signal Transduction mechanisms	2	2.0%	1	50%
U	Intracellular trafficking, secretion, and vesicular transport	2	2.0%	2	100%
V	Defense Mechanisms	2	2.0%	2	100%
	No COG assigned	14	13.9%	1	7%

[1] Count and % sum to greater than 101 and 100%, respectively, as a small number of proteins were assigned to more than one COG category.

REFERENCES

1. Levy AT. 2016. Insights into elemental sulfur metabolism and low-energy adaptations in *Chlorobaculum tepidum* to enable biotechnology based processes for waste sulfur utilization. Ph.D. Dissertation, University of Delaware, Newark, DE, USA.

2. Hanson TE, Bonsu E, Tuerk A, Marnocha CL, Powell DH, Chan CS. 2016. *Chlorobaculum tepidum* growth on biogenic S(0) as the sole photosynthetic electron donor. Environ Microbiol 18:2856–2867.

3. Jang K-S, Sweredoski MJ, Graham RLJ, Hess S, Clemons WM Jr. 2014. Comprehensive proteomic profiling of outer membrane vesicles from *Campylobacter jejuni*. J Proteomics 98:90–98.

4. Lee E-Y, Bang JY, Park GW, Choi D-S, Kang JS, Kim H-J, Park K-S, Lee J-O, Kim Y-K, Kwon K-H, Kim K-P, Gho YS. 2007. Global proteomic profiling of native outer membrane vesicles derived from *Escherichia coli*. Proteomics 7:3143–3153.

5. Galka F, Wai SN, Kusch H, Engelmann S, Hecker M, Schmeck B, Hippenstiel S, Uhlin BE, Steinert M. 2008. Proteomic characterization of the whole secretome of *Legionella pneumophila* and functional analysis of outer membrane vesicles. Infect Immun 76:1825–1836.

6. Whitworth DE, Slade SE, Mironas A. 2015. Composition of distinct sub-proteomes in *Myxococcus xanthus*: metabolic cost and amino acid availability. Amino Acids 47:2521–2531.

7. Post DMB, Zhang D, Eastvold JS, Teghanemt A, Gibson BW, Weiss JP. 2005. Biochemical and functional characterization of membrane blebs purified from *Neisseria meningitidis* serogroup B. J Biol Chem 280:38383–38394.

8. Lappann M, Otto A, Becher D, Vogel U. 2013. Comparative proteome analysis of spontaneous outer membrane vesicles and purified outer membranes of *Neisseria meningitidis*. J Bacteriol 195:4425–4435.

9. Ballok AE, Filkins LM, Bomberger JM, Stanton BA, O'Toole GA. 2014. Epoxide-mediated differential packaging of Cif and other virulence factors into outer membrane vesicles. J Bacteriol 196:3633–3642.

10. Choi D-S, Kim D-K, Choi SJ, Lee J, Choi J-P, Rho S, Park S-H, Kim Y-K, Hwang D, Gho YS. 2011. Proteomic analysis of outer membrane vesicles derived from *Pseudomonas aeruginosa*. Proteomics 11:3424–3429.

11. Couto N, Schooling SR, Dutcher JR, Barber J. 2015. Proteome profiles of outer membrane vesicles and extracellular matrix of *Pseudomonas aeruginosa* biofilms. J Proteome Res 14:4207–4222.

12. Bai J, Kim SI, Ryu S, Yoon H. 2014. Identification and characterization of outer membrane vesicle-associated proteins in *Salmonella enterica* serovar Typhimurium. Infect Immun 82:4001–4010.

13. Altindis E, Fu Y, Mekalanos JJ. 2014. Proteomic analysis of *Vibrio cholerae* outer membrane vesicles. Proc Natl Acad Sci U S A 111:E1548-56.
